# The Importance of Immunological Disorders in the Pathogenesis of Lichen Sclerosus in Pediatric Patients: A Systematic Review

**DOI:** 10.3390/ijms232214212

**Published:** 2022-11-17

**Authors:** Anna Torres, Monika Zaborek-Łyczba, Jakub Łyczba, Paulina Mertowska, Sebastian Mertowski, Ewelina Grywalska

**Affiliations:** 1 Department of Pediatric and Adolescent Gynecology, Medical University of Lublin, 20-093 Lublin, Poland; 2 Department of Experimental Immunology, Medical University of Lublin, 20-093 Lublin, Poland

**Keywords:** lichen sclerosus, lichen sclerosus vulva, pediatric patient, adolescent, immunopathogenesis, immune system, immunogenetics

## Abstract

Lichen sclerosus (LS) is defined as a chronic mucocutaneous inflammatory disease with a localization predominantly to the anus and genitals (vulvar sclerosus (VLS)). Pediatric lichen sclerosus (LS) is a chronic inflammatory skin condition with predilection for the anogenital area that if untreated can lead to scarring. Vulvar LS is characterized by two peaks in incidence: it occurs in prepubertal girls and in postmenopausal women. To date, several mechanisms and risk factors have been proposed in the pathogenesis of pediatric vulvar LS; however, the etiology of this condition is still not fully understood and constitutes a challenge for scientists and clinicians. The presented research aimed to systematically review the existing literature on the pathogenesis of pediatric LS and to identify possible underlying autoimmune mechanisms and molecular networks. The clinical presentation of pediatric lichen sclerosus and available treatment modalities are also presented to acquaint a broader audience with this underdiagnosed and undertreated condition. As a result of our review, we discuss several potential mechanisms, molecules, and pathways that have been recognized in this disease. The purpose of our review was also to summarize what we can induce in further studies, which will ultimately help to identify the mechanism responsible for the disease and aid in the development of new, more effective treatment strategies for diagnosis and treatment by clinicians and researchers.

## 1. Introduction

Lichen sclerosus (LS) is defined as a mucocutaneous chronic inflammatory disease with a predilection to the anogenital area [1,2]. It has the potential to cause atrophy, destructive scarring, impaired function, and malignant evolution. Scars from lichen sclerosus of the vulva are more common in adults than in children [3]. The risk of vulvar squamous cell carcinoma is well documented in adult women, but because children present less scarring, the risk of cancer in childhood lichen sclerosus remains unknown [4]. LS is more common in women and has a bimodal peak incidence in premenstrual girls and menopausal women [5,6]. A study by Maronna et al. in 2004 [7] involving 327 patients with LS showed that the mean age of onset of this type of disease was 5.4 years in girls and 55.1 years in women. As indicated in the literature, the prevalence of LS ranges from 1:70 to 1:1000 in women and 1:900 in children [8]. Delayed diagnosis is not uncommon in girls with LS; the mean time to diagnosis is 1 to 1.6 years [9,10]. Vulval lichen sclerosus (VLS) that develops during childhood or adolescence is then referred to as juvenile VLS (JVLS). VLS is one of the most common diagnoses in girls and has persistent genital symptoms including itching, pain, dysuria, and constipation [11]. In the case of pediatric patients with chronic vulvar complaints (itching, pain, painful urination, constipation), VLS is one of the most common diagnoses [8,12]. Clinical signs of JVLS, as in adults, include pallor, white plaques, purpura, furrows, loss of architecture, and narrowing of the vaginal opening. Possible long-term consequences of JVLS include the persistence of symptomatic disease; anatomical changes; potential risk of developing vulvar squamous cell carcinoma (VSCC); and detrimental effects on sexuality, obstetric outcome, self-image, and other aspects of quality of life [12]. Histologically, LS is characterized by a band-like lymphocytic infiltrate and a thinned epidermis with vacuole changes in the basal layer. In long-term classic LS, the lymphocytic infiltration is located under the homogenized collagen band below the dermoepidermal junction [13]. While there is increasing evidence that autoimmune mechanisms play a key role in the pathogenesis and progression of this type of disease, it is still not adequately understood [14,15,16]. 

Therefore, the aim of this publication was to conduct a systematic review of the literature and investigate the pathogenesis of VLS by identifying diverse immune disorders and associated molecular networks. Additionally, we wanted to present information on the functional role of selected molecules in terms of their potential clinical utility in the diagnosis and treatment of LS.

## 2. Materials and Methods

### 2.1. Search Strategy, Study Selection, and Data Extraction

#### 2.1.1. Study Condition and Participants

Lichen sclerosus (LS) is a chronic, progressive, inflammatory skin disease that most commonly affects the anus and genitals of women, but also of young adults and children.

The subjects/population studied were people with lichen sclerosus (diagnosed according to recognized diagnostic guidelines); one of the primary criteria for inclusion in the analysis was gender (female) and age (children and/or young adults).

Therefore, in this article we set a goal of addressing three quite important issues: (1) the course and epidemiology of LS/VLS in pediatric patients and young adults; (2) how the immune system is involved in the development of LS/VLS progression in this group of patients; and (3) whether there are any immunogenetic determinants for the development and progression of LS/VLS in pediatric and young adult patients.

#### 2.1.2. Databases and Search Strategy

**Figure 1:**
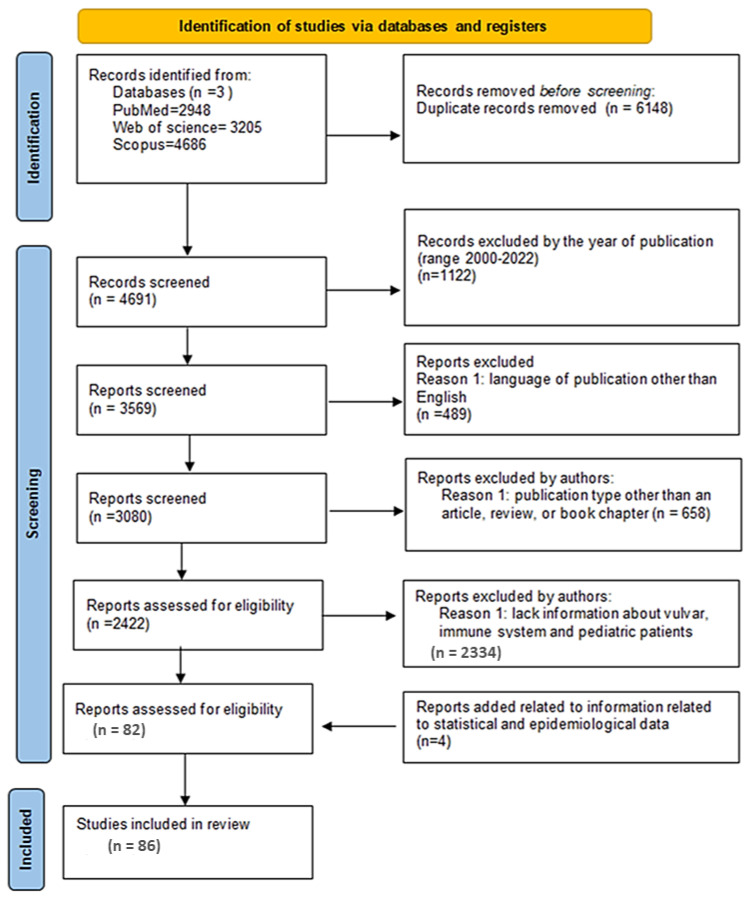
PRISMA flow chart illustrating the selection process for articles for this review.

A systematic review of the literature was carried out in accordance with the Preferred Reporting Items for Systematic Reviews and Meta-Analyses (PRISMA) guidelines. The database analysis (using PubMed, Web of Science, Scopus, EBSCO, PsycINFO, and the Social Science Citation Index) was carried out by two researchers (M.Z.-Ł. and J.Ł.). Due to having the highest hit rates, the first three databases were used for further analysis. The analysis of the literature was carried out based on the determination of the time range of literature items (2000–2022) and an analysis of the presence of the following keywords: ‘Lichen sclerosus’, ‘Vulval lichen sclerosus, ‘pediatric’, ‘juvenile’, and ‘immune system’. Then the publications were filtered in terms of document type and publication language. Other literature items concerned symptoms, risk factors, immunopathogenesis, and immunogenetic determinants of LS/VLS in pediatric patients and young adults. The detailed literature search strategy is shown in Figure 1.

#### 2.1.3. Data Extraction, Bias Risk Assessment, and Strategy for Data Synthesis

Following the removal of duplicates, the titles and abstracts were reviewed by two researchers (P.M. and S.M.) in accordance with the PRISMA 2020 for Abstracts Checklist, and all disputes regarding the inclusion of publications in the research were resolved through discussion with a third independent researcher (A.T.). Then, two researchers (P.M. and S.M.) independently extracted the data using a template in Microsoft Excel©. The following data were collected: (1) epidemiological data of LS/VLS in pediatric and young adult patients; (2) LS/VLS symptoms and risk factors; (3) research on changes in the immune system of patients with LS/VLS; and (4) information on the genetic determinants of changes in the immune system and its components. The bias risk assessment was performed by an independent researcher (E.G.) based on the PRISMA 2020 statement containing a checklist for the introduction, methods, results, and discussion sections of the systematic review report. The strategy for data synthesis was carried out while taking into account the following steps: deciding what was relevant; reading the studies; determining how the studies were related to each other; translating the studies into each other; synthesizing the translations; and expressing the synthesis.

## 3. Results and Discussion

### 3.1. Characteristics of Lichen Sclerosus of the Vulva

#### 3.1.1. Epidemiology

The exact number of people with LS and the incidence of is the disease are not exactly known and are difficult to estimate, and they are possibly underestimated. Many cases are diagnosed incorrectly or are asymptomatic [17]. Moreover, some patients consider the symptoms of the disease to be embarrassing and do not see their doctors at all [18]. The results of epidemiological studies are inconsistent in the reporting the prevalence data. Research by Goldstein et al. in 2005 [19] showed that the prevalence of LS in general gynecological practice was as high as 1.7%. On the other hand, a study from 2020 performed by the team of Melnick et al. estimated the prevalence of the disease to be 0.1% in the general US population and only 0.01% in the pediatric subgroup (patients under 18 years of age) [5]. Different results were presented by Powell and Wojnarowska [8], who described the prevalence of LS in prepubertal girls as 0.1%. 

The literature data show that most of the diagnoses were made by dermatologists or gynecologists, but the patients visited other specialists as well, including general practitioners (GPs) and internists [20]. The results of this study confirmed that LS could indeed be underestimated and underdiagnosed, especially in younger women [21,22]. Moreover, the prevalence of VLS in adolescent girls is underestimated due to the incorrect recognition of symptoms by GPs and delayed access to specialists in pediatric gynecology or dermatology [8]. A study conducted in the Netherlands by Bleeker et al. in 2016 [23] showed an increase in the incidence of LS between 1991 and 2011 from 7.4 to 14.6 per 100,000 women. The mean age of onset in girls with VLS was 7.1 years, while the mean delay from symptom onset to diagnosis was 1.3 years. That delay was mainly due to a misdiagnosis prior to referral to a pediatric gynecology or dermatology clinic [21,22,23,24]. Another study by Powell and Wojnarowska [8] concerning 70 cases of LS in girls indicated that the mean age at onset of symptoms was 5.0 years (range 1–12 years), and the mean age at diagnosis was 6.7 years (range 3–14 years). In another study by Ismail and Owen [25] from 2019, the median age at onset of LS symptoms was 5 years (age range 2–8.5 years), and the median age at diagnosis of MD was 8 years.

#### 3.1.2. Risk Factors

**Figure 2:**
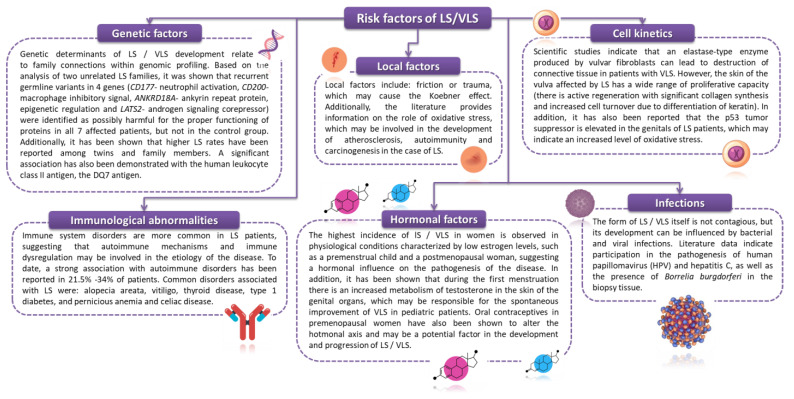
Risk factors for the occurrence of LS/VLS based on [26,27,28,29,30,31,32,33,34,35,36].

The etiology of LS and VLS is not fully understood and is a challenge for many modern scientists and doctors. Currently, based on the epidemiological data available in the literature, a number of mechanisms/risk factors involved in the pathogenesis of LS have been proposed. This applies to the presence of genetic and infectious factors as well as local factors, hormonal factors, and immunological abnormalities (Figure 2) [26,27,28,29,30,31,32,33,34,35,36]. 

#### 3.1.3. LS/VLS Diagnostic Challenges

So far, it has not been possible to characterize the etiological cause and pathogenesis of the development of LS/VLS. In the early stages of LS, not only the symptoms but also the histological picture may be atypical [37]. The histological features in early LS are quite subtle and may overlap with those seen in diseases such as psoriasis or lichen planus, including hyperkeratosis of the lumen and hypergranulosis of the structures of the appendages. Early skin lesions include subepithelial edema, homogenized collagen, and dilated blood vessels directly beneath the basement membrane. Lymphocytic infiltration may be lichenoid or interstitial with exocytosis of epidermal lymphocytes and lymphocytic/lymphohistiocytic vasculitis [38,39]. The classic histological features of more advanced uncomplicated LS are hyperkeratosis, epidermal atrophy with flattening of the retinal ridges, changes in the vacuolar interface, loss of elastic fibers, and hyalinization of the lamina propria with underlying lymphocytic infiltration, but the feature may be prominent acanthosis. especially in vulvar LS [40]. One of the largest analyses of VLS histopathological features in the adolescent population was described by Morell et al. [41]. That study included biopsies of 100 teenage cases of vulvovaginitis and analyzed the presence or absence of the most important histopathological features of thVLS. Histological investigations have shown that hyperkeratosis, sclerosis of homogenized collagen/skin, and substantial vacuolation of epidermal keratinocytes almost always occurred; epidermal atrophy was present in half of the cases. Moderate to deep perivascular infiltration and ectatic vessels were observed in three-quarters of the subjects. As for the appendages, analysis of the hair follicles showed perifollicular dermatitis in more than half of the cases and obstruction of the hair follicles in one-third of the cases. Nerves and sweat glands were seen frequently; however, a minority of cases showed specific changes attributable to VLS. The interstitial infiltrate observed in the majority of cases was moderate to deep in 80% of cases, although most of it was superficial. Most of the assessed histopathological features did not differ statistically from the period before or after puberty. Hair follicles and sweat glands were more common in biopsies of people in the prepubertal period. Taken together, the authors concluded that a number of histopathological features known to adult LS were also present in juvenile LS vulva even at a very young age, including histopathological features associated with autoimmune disease, which supported the idea of a similar pathogenesis [41].

#### 3.1.4. LS/VLS Symptoms

Symptoms and signs of VLS are very diverse and may be nonspecific or discrete [37]. Therefore, the correct diagnosis may be difficult and is often delayed by at least one to two years [21].

The most common symptoms of VLS are vulvar itching (70–86%), soreness and burning (80%), bleeding (61%), dysuria and fear of voiding (43%), as well as constipation and painful defecation (11%). Excessive rubbing of the genitals can damage skin, which results in bleeding. Excoriations resulting from rubbing often become infected, leading to secondary symptoms related to bacterial or fungal vulvitis, which may obscure the typical signs and delay the correct diagnosis in about one-third of girls with VLS [21].

The noteworthy characteristic of JVLS is that its symptoms are not always proportional to the extent of signs present on the vulva. Patients with minor vulvar changes may present with severe pruritus, whereas those with severe lesions often do not complain of any symptoms. It has been reported that approximately 7% of girls are asymptomatic but still have significant findings noted upon physical examination [21,42,43,44]. 

Skin manifestations of VLS are multifarious and range from discrete white patches or longitudinal fissures that often are limited to interlabial sulci to the typical, wide-spread changes described below. The lesions can affect the labia minora and majora, the clitoral hood, the clitoris, the perineum, and the area around the anus, whereas the vaginal mucosa is usually spared [45,46].

The classic presentation of advanced VLS includes clearly demarcated, ivory-white skin with a parchment-like or cigarette-paper appearance (91%); the lesion has a characteristic hourglass or “figure of eight” shape that involves the labia majora and minora as well as the skin around the anus [29,36]. In a study by Ismail and Owen, the most common clinical sign presented was anogenital pallor, which occurred in 23 out of 26 girls [25]. This classic skin appearance can be accompanied by skin fissures and ruptures, ecchymoses, subepithelial hemorrhage, and superficial erosions (73%) or telangiectasias. Sometimes the skin can be thickened due to hyperkeratosis as a result of repeated excoriations (52%) [29,36,47]. 

On the other hand, the signs of early LS are often quite discrete and can be easily missed especially in patients with light skin color. These early signs include poorly demarcated delicate whiting of the skin and small ivory-white or rose-colored patches often accompanied by single vertical fissures most commonly discovered just above the clitoris or within the interlabial sulci. Importantly, the changes can be unilateral and can sometimes come and go with the use of over-the-counter topical emollients [9,46,47,48,49,50].

Extragenital lesions can coexist and were reported in 18% of children with VLS. They may appear anywhere on the body and present as white, flat lumps that often merge into larger foci with a shiny porcelain surface that sometimes is surrounded by a purple halo [51]. 

Scarring and changes in the vulvar architecture are rare before puberty; however, in untreated LS, the chronic sequel includes both anatomical and functional adverse effects. A distorted vulvar architecture includes adhesions and resorptions of the labia, clitoral phimosis with pseudocysts, scarification, and narrowed vaginal introitus. These anatomic changes may lead to chronic pain, dyspareunia, problems with urination and defecation, and obstetrical complications [29]. 

### 3.2. Immunopathogenesis of LS/VLS

**Figure 3:**
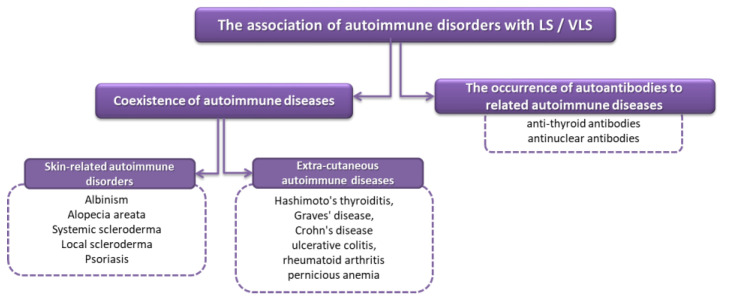
Autoimmune links with LS/VLS based on [16,52].

The etiology of LS has not yet been sufficiently elucidated, but increasing evidence suggests that autoimmune mechanisms play a key pathogenetic role; the available literature data emphasizes the role of immune factors. The literature data show that LS/VLS coexists in 15–34% of cases in adult women and 14% in girls with allergies or autoimmune diseases such as vitiligo, thyroiditis, type 1 diabetes, or alopecia areata [16,52]. The autoimmune basis that accompanies LS includes not only the presence of cocutaneous autoimmune diseases, but also their extracutaneous variants and the presence of autoantibodies to related autoimmune diseases (Figure 3).

**Figure 4:**
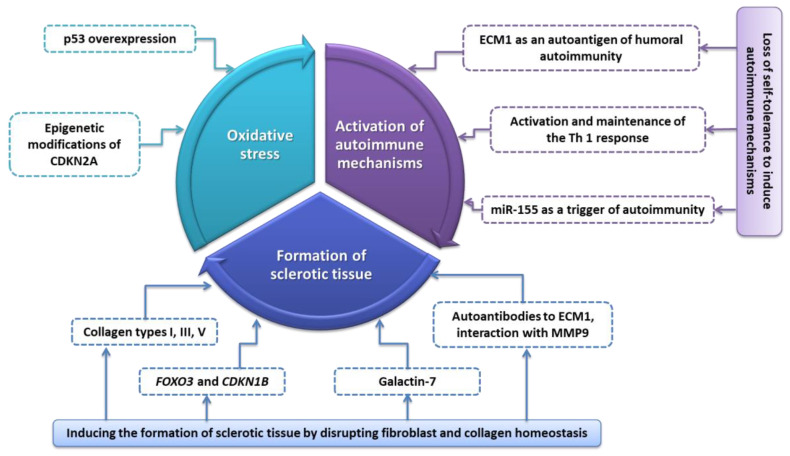
Schematic representation of the three major mechanisms involved in LS/VLS pathogenesis based on [14]. ECM1—extracellular matrix protein 1; CDKN1B—cyclin-dependent kinase inhibitor 1B; FOXO3—forkhead box O3; CDKN2A—cyclin-dependent kinase inhibitor 2A; MMP9—matrix metallopeptidase 9; p53—protein 53.

According to previous studies, the most common autoimmune diseases associated with LS were autoimmune thyroiditis (12%), alopecia areata (9%), vitiligo (6%), and pernicious anemia (2%) [16,52]. The research conducted by the team of Cooper et al. [53] showed that 28% of women diagnosed with LS were also diagnosed with one or more related autoimmune diseases, which was more than a 3-fold increase compared to patients in the control group. However, despite the fact that scientists have shown a strong relationship between LS and autoimmune disease, the etiology of this disease remains the subject of much intense research. In recent years, three mechanisms have been proposed that, through interactions, can lead to the development and progression of LS/VLS. These include autoimmune mechanisms, sclerotic tissue formation, and the triggering of oxidative stress (Figure 4).

**Figure 5:**
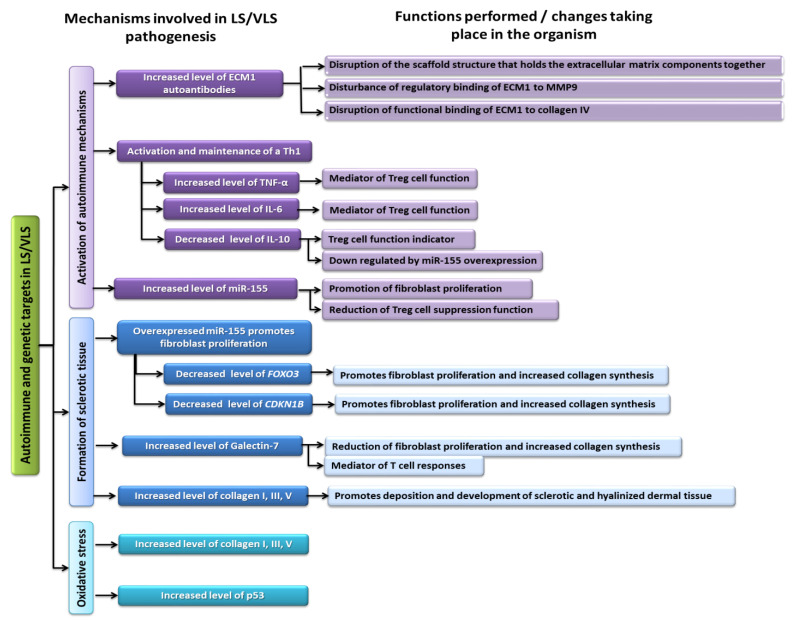
Schematic presentation of LS autoimmune and genetic targets based on [16,60]. ECM1—extracellular matrix protein 1; CDKN1B—cyclin-dependent kinase inhibitor 1B; FOXO3—forkhead box O3; CDKN2A—cyclin-dependent kinase inhibitor 2A; MMP9—matrix metallopeptidase 9; p53—protein 53.

Within the first mechanism, we can distinguish three characteristic processes that lead to a loss in self-tolerance to induce autoimmune mechanisms: dysfunction of extracellular matrix protein 1 (ECM1) as an autoantigen of humoral autoimmunity; activation and maintenance of the Th 1 response; and the role of miR-155. The ECM1 protein (which is a soluble glycoprotein) is a specific type of scaffold for many extracellular components such as laminin 332, laminin 10, collagen IV, fibulin polysaccharides, proteoglycans, phospholipids, and proteolytic enzymes (MM9) [54]. The ECM1 glycoprotein has several important biological functions. In the epidermis, ECM1 plays a role in the control of keratinocyte differentiation; in the dermis it plays a role in the structural organization of the dermis through binding to perlekan, matrix metalloproteinase-9, and fibulin. A reduction in matrix metallopeptidase 9 (MMP9) activity by ECM1 is of particular importance for histological changes [55]. Its potential involvement in the process of LS pathogenesis was evidenced by a study by the Oyama team in 2003 [56] in which researchers found that circulating IgG autoantibodies targeting extracellular matrix protein 1 (ECM1) were found in the serum of 74% of women with LS of the anus and genitals compared with 7% in the control group. While these autoantibodies were not expected to be pathogenic to LS, their presence suggested further evidence that autoimmune mechanisms may be involved in the etiology of the disease. ECM1 autoreactivity was more likely in subjects with disease duration greater than 1 year and/or in subjects with more extensive disease, suggesting that ECM1 autoreactivity may be involved in disease progression rather than disease initiation [54,57]. The ECM1 glycoprotein has several important biological functions. Fujimoto et al. found specific autoantibodies in LS patients that targeted the c-terminal second tandem repeat (exon 7) of recombinant ECM1, the same region to which MMP9 also binds [54]. Disruption of normal regulatory binding of ECM1 to MMP9 may be caused by a loss of this region either by mutation of the ECM1 gene or by autoantibodies to that particular region of ECM1, resulting in overactive MMP9 collagenase activity that disrupts collagen homeostasis, which in turn may explain the focal basement membrane damage seen in LS [54]. Due to the existence of heterogeneity within ECM1 antibodies targeting different ECM1 epitopes, the authors speculated that the anti-ECM1 antibodies in LS mainly influenced the functional binding of ECM1 to collagen IV and perlekan more than MMP9 (Figure 5) [56,58]. Thickening can occur instead of degradation in other regions of the basement membrane. Such a seemingly paradoxical phenomenon may also be due to the hyperactivity of MMP9, which in addition to deregulation of binding to ECM1 provided by its collagenase function, also cleaves latent transforming growth factor beta (TGF-β). This effect is a probable mechanism of TGF-β activation, which enhances collagen synthesis and regeneration and is visible in lipid proteinosis (LiP) [54]. Another process involves activating and maintaining a Th1 response in the course of LS/VLS. In LS patients, a significant increase in the number of CD8+ T cells and Treg cells was observed, while the number of CD4+ T cells in LS was not significantly increased. These data were consistent with published studies that described dense T-cell infiltration in LS [59]. To investigate whether T cells are involved in the Th1 or Th2 response, Terlou et al. [60] studied various chemokine receptors. Th1 cells express the C-X-C motif chemokine receptor 3 (CXCR3 and C-C chemokine receptor type 5 (CCR5), while Th2 cells express CCR3 and CCR4. Scientists found high levels of CXCR3 and CCR5 in LS and no expression of the C-C chemokine receptor type 3 (CCR3) or C-C chemokine receptor type 4 (CCR4), indicating infiltration of Th1 cells. However, the role of the Th1 response in autoimmune disorders is not fully understood and appears to be important in the induction, maintenance, and exacerbation of chronic inflammation [61]. This is evidenced by the production of IFNγ by T-helper cells (CD4 + T cells). This interferon is one of the key factors in the Th1 response. In previous studies, it was found that INFy mRNA was strongly elevated in LS. In addition to IFN-γ, researchers found elevated levels of other proinflammatory cytokines in LS (in particular, IL-1, IL-7, IL-15, and TNF-α) compared to anti-inflammatory cytokines (TNF-β and IL-10), which suggested the involvement of Th1 lymphocytes in the development of LS (Figure 4) [60,62].

The third process involves the operation of miR-155 (Figure 5). MicroRNAs (miR-155) are endogenous RNAs that act as regulators of gene expression by binding to the 3’ untranslated region (3′ UTR) of a target mRNA transcript [63,64]. The abnormal level of microRNA expression may be thus associated with the pathogenesis of LS. MiR-155 has been shown to be expressed in activated immune cells (i.e., macrophages, dendritic cells, B cells, and T cells) and plays an important regulatory role in the production of cytokines, chemokines, and transcription factors [60]. miR-155 is also involved in promoting Th1 differentiation [60]. When overexpressed, miR-155 can interfere with regulatory T cell (Treg)-mediated suppression, inducing a loss of self-tolerance and promoting inflammation, thereby inducing autoimmunity [16]. Additionally, miR-155 inhibits the tumor suppressor genes *FOXO3* and *CDKN1B*, which leads to the final synthesis of collagen. In addition, researchers demonstrated the presence of circulating autoantibodies against basement membrane zone (BMZ) components (mainly BP180/collagen XVII and BP230) in one-third of vulvar LS patients (Figure 5) [57]. Among 43% of patients with LS of the vulva in the study by Baldo et al. [65], the NC16A domain of BP180 was shown to be a target for circulating T cells with associated anti-BP180 autoantibodies. In another study, the authors found an incidence of >40% (4/9) of circulating anti-BMZ autoantibodies in LS girls [66]. Three children showed IgG BMZ antibodies in immunofluorescence and none had IgA BMZ autoantibodies. These results suggested specific immunoreactivity against the BMZ BP180/collagen XVII antigen. This confirmed that childhood LS shares etiological factors with adult disease despite the difference in age of onset and suggests that autoimmunity in this region is important in VLS [66]. Inflammation of the Th1 cytokine environment leads to the release of reactive oxygen species (ROS), which promotes autoimmunity and oxidative stress [16]. Oxidative stress contributes to the inactivation of tumor suppressor genes, including p53 and CDKN2A, which leads to cell proliferation and conversion to malignancy (Figure 5). In addition, inflammation and release of ROS, vascular changes caused by hardening, and hyalinization of the skin lead to the development of atherosclerotic vessels that restrict oxygen flow and induce ischemic stress. Such stress in turn increases the stability and accumulation of p53, possibly as a compensatory mechanism to counteract the harmful effects of oxidative stress [67,68].

### 3.3. Immunogenetic Conditions of LS/VLS

Scientists have also suggested a potential role of genetic predisposition. Family studies showed a positive history of LS in the family [69,70]. In one of the large observational cohort studies involving a total of 1052 women with vulvovaginitis, it was found that 12% had a positive family history of ZL, which indicated family cases of LS [28]. The genetic component was further confirmed in articles on LS case reports for monozygotic and dizygotic twins, although the mode of inheritance has not yet been established [71,72]. The hypothesis of genetic susceptibility was supported by studies that indicated a significant association of LS with genes regulating human leukocyte antigen (HLA) class II antigens, which are involved in humoral immunity. Many studies focused on HLA DQ7, which has been shown to be more common in 50% of adult women and 66% of prepubertal women compared to 31% in the control group [73,74]. In addition, Powell et al. [73] found that 16% of children were homozygous for DQ7 as opposed to 5% of the controls. In the childhood group, only 4% had other autoimmune diseases, but 56% of their parents or grandparents did. Age differences made comparison difficult, but family history of autoimmunity appeared to be strong in the early-onset group, in addition to a stronger association with DQ7 [73]. It was shown that HLA-DR12 HLA DQ8, DQ9, and haplotype DRB1*12/DQB1*0301/04/09/010 appeared more often in LS patients than in the control group [28,74]. In contrast, HLA-DR17 showed a negative relationship with LS, which suggests protective properties [74]. The associated haplotypes of these specific HLA antigens may play a role in LS compliance and protection [16,74]. Considering autoimmune diseases with LS, DRB1*13 appears to be more prominent in people with LS alone than in people with both LS and autoimmune disease. Such data suggest that although DRB1*13 may not protect against LS, it may protect against the coexistence of LS and the onset of autoimmune disease [74].

### 3.4. Treatment Used in the Course of LS/VLS

The course of lichen sclerosus is characterized by relapses and remissions. The main goal of VLS treatment is to alleviate clinical symptoms; reduce the signs; and prevent complications such as scarring, adhesions, and atrophic changes [75]. To date, three modes of treatment have been found effective in LS/VLS: topical glucocorticosteroids, calcineurin inhibitors, and retinoids [15,76,77,78].

**Figure 6:**
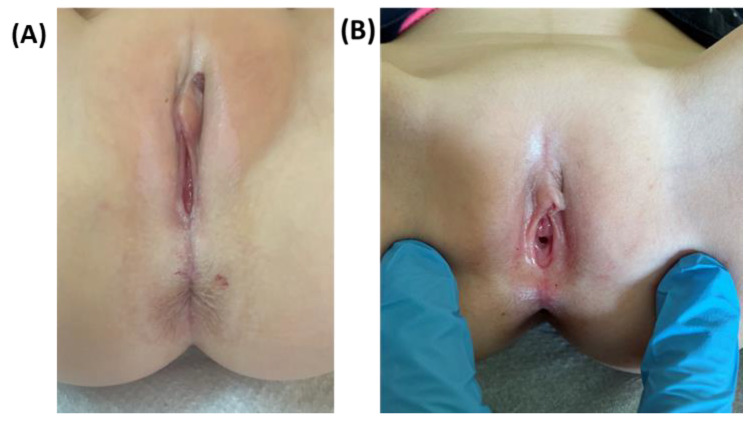
Sample image of premenarchal lichen. (**A**) Typical presentation of pediatric lichen sclerosus in a 5-year old girl; (**B**) the same case after 8 weeks of treatment with 0.05% clobetasol propionate.

The first-line therapy in adults and children, which consists of ultrapotent topical corticosteroids (UPTC), is recommended by both European and international guidelines [48,75,79]. The most often described treatment regimen, which is based on the use of 0.05% clobetasol propionate ointment for 6 to 12 weeks, is called induction treatment (Figure 6) [80]. 

Randomized trials were not available in children and adolescents; data were extrapolated from randomized studies performed in adults as well as from retrospective studies and case series in children [75]. The authors defined the maximum allowable dose of clobetasol propionate to be approximately 10 g per month [75,80,81,82].

The European guidelines reported that treatment with 0.05% clobetasol propionate reduced symptoms and showed signs of improvement in 65–100% of girls; in 20–70%, such treatment was associated with complete remission without the need for maintenance treatment [33]. Nevertheless, follow-up visits should be scheduled in all patients at intervals of 3 to 6 months because asymptomatic relapses may occur and result in irreversible scarring of vulvar structures. After the initial six months, annual follow-ups should be scheduled for two subsequent years regardless of whether the need for maintenance therapy persists [75]. 

Steroid injections were proposed as a treatment option for adult patients with a treatment-resistant itch or who were unable to apply topical steroids [83].

The European guidelines emphasize the role of calcineurin inhibitors as an alternative to topical corticosteroids in LS [33]. According to these guidelines, tacrolimus ointment is an effective and safe form of VLS therapy in adults and is recommended for the use as a second-line therapy or a maintenance treatment. The rationale for the use of calcineurine inhibitors in the treatment of VLS consists of theoretical benefits resulting from a lesser systemic and local adverse influence as compared to topical UPCT. Both calcineurin inhibitors (pimecrolimus and tacrolimus) are covered by an FDA black box warning due to a possible causal relationship between the long-term use of topical calcineurin inhibitors and the development of skin cancer and lymphoma [84]. Due to this warning and the well-known efficacy of potent steroids, both pimecrolimus and tacrolimus should be considered as second-line agents [75].

The local action of retinoids can be explained by their influence on the increase in CD44 expression in the epidermis. In contrast, studies indicated that CD44 expression of the epidermis was reduced or absent in VLS lesions. Studies have shown a reduction in symptoms such as itching, burning, and pain when using topical retinoids. The European guidelines for the treatment of VLS indicate that retinoids are not recommended for monotherapy but rather as an adjunctive treatment during topical UPTC therapy. Additionally, the guidelines emphasize that oral therapy is not recommended due to numerous side effects [15,48]. Studies did not confirm the efficacy of topical testosterone, dihydrotestosterone, or progesterone; these substances are not recommended to treat VLS in adults or children [85]. Multiple other therapeutics were described in single trials in adult VLS; these include UVA1 phototherapy, photodynamic therapy, oral cyclosporine, and oral methotrexate. Cryotherapy, laser therapy with carbon dioxide or an erbium-doped yttrium aluminum garnet (Er:YAG) laser, and focused ultrasound have also been used with varying efficacy. Using adipose-derived mesenchymal stem cells and platelet-rich plasma is a novel promising approach [80,86].

The chance for complete and long-standing remissions with well-studied treatment options is possible and seems to be higher in children than in adults; however, maintenance therapy, which prevents the long-term sequalae, was recommended by most authors, with UPTC administered once to twice a week along with topical emollients [75].

## 4. Conclusions

The etiology of LS/VLS is not fully understood and is a challenge for many scientists and doctors in their daily practice. To date, several mechanisms and risk factors in the pathogenesis of LS have been proposed, including genetic predisposition, infectious agents, endocrine disruptions, and immune disorders. The analyses reviewed in this publication showed that the pathogenesis of VLS may be conditioned by various disorders of the immune system and related molecular networks in pediatric patients and young adult women. The immunopathogenesis of LS/VLS in pediatric patients is associated with autoimmune disorders (the coexistence of autoimmune diseases and/or the presence of autoantibodies to autoimmune-related diseases). Additional factors that also affect the pathogenesis of LS/VLS are inflammation, oxidative stress, and vascular changes caused by skin hardening and hyalinization, which lead to the development of atherosclerotic vessels. Unfortunately, despite the collection of information on the functional role of selected molecules involved in the pathogenesis of LS/VLS, we found no significant data on their potential clinical usefulness in the diagnosis of this type of disease. We strongly believe that this publication will draw the attention of doctors, diagnosticians, and scientists to this extremely important research problem and will allow for the development of further interdisciplinary and multicenter research, which will enable the identification and verification of genes, proteins, or glycoproteins that may serve as LS/VLS biomarkers in the future.

## Data Availability

The data presented in this study are available on request from the authors.
